# Multivariate simulation framework reveals performance of multi-trait GWAS methods

**DOI:** 10.1038/srep38837

**Published:** 2017-03-13

**Authors:** Heather F. Porter, Paul F. O’Reilly

**Affiliations:** 1 MRC SGDP Centre, Institute of Psychiatry, Psychology & Neuroscience, King’s College London, London, SE5 8AF, UK

## Abstract

Burgeoning availability of genome-wide association study (GWAS) results and national biobank data has led to growing interest in performing multi-trait genetic analyses. Numerous multi-trait GWAS methods that exploit either summary statistics or individual-level data have been developed, but their relative performance is unclear. Here we develop a simulation framework to model the complex networks underlying multivariate genetic epidemiology, enabling the vast model space of genetic effects on multiple correlated traits to be explored systematically. We perform a comprehensive comparison of the leading multi-trait GWAS methods, finding: (1) method performance is highly sensitive to the specific combination of genetic effects and phenotypic correlations, (2) most of the current multivariate methods have remarkably similar statistical power, and (3) multivariate methods may offer a substantial increase in the discovery of genetic variants over the standard univariate approach. We believe our findings offer the clearest picture to date of the relative performance of multi-trait GWAS methods and act as a guide for method selection. We provide a web application and open-source software program implementing our simulation framework, for: (i) further benchmarking of multivariate GWAS methods, (ii) power calculations for multivariate genetic studies, and (iii) generating data for testing any multivariate method in genetic epidemiology.

The early stages of the GWAS era were dominated by studies with a single phenotype as outcome^1^,^2^,^3^, while in recent years multi-trait analyses have become more popular^4^,^5^,^6^. Multivariate methods have been developed to increase statistical power and identify pleiotropic loci in GWAS^7^,^8^,^9^,^10^,^11^,^12^,^13^,^14^,^15^,^16^,^17^,^18^,^19^,^20^, while polygenic risk score and co-heritability estimation methods are now routinely applied to GWAS data to assess shared genetic aetiology across multiple traits^4^,^21^,^22^,^23^,^24^. However, these methods have been developed and applied in the absence of a dedicated simulation framework for generating data reflective of the complexity of multivariate data. Here we present a simulation framework designed to capture as much of the multivariate data landscape as possible, allowing researchers to benchmark methods across a range of simulation scenarios of genetic variants affecting multiple traits. We also incorporate real data so that simulated genetic effects and phenotypic correlations can closely match reality.

We exploit our simulation framework to perform a comprehensive comparison of the current leading multi-trait GWAS methods. While some methods simultaneously model multiple SNPs and multiple traits^7^,^8^,^20^, we focus on the more common single-SNP methods to isolate the methodological advances responsible for the greatest increases in power when modelling multiple phenotypes. We compare 10 methods: min-*P*^9^, TATES^10^, S_Hom_^11^, S_Het_^11^, MANOVA, CCA^12^ (mv-PLINK), Combined-PC^13^, MultiPhen^9^, mv-BIMBAM^14^ and mv-SNPTEST^15^ (see Methods and Supplementary Table 1). Benchmarking of these methods has so far been limited to a small number of modelling scenarios, comparing few methods and testing a small number of traits^9^,^10^,^11^,^12^,^13^,^14^,^25^. The illustration of method performance is often challenging to interpret and is highly inconsistent across publications. Researchers are thus left with a perplexing choice between competing methods. Our dedicated simulation framework enables a systematic and rigorous search through the multivariate model space. We present results across a range of genetic effects and phenotypic correlations, from which a clear picture of the relative performance of the methods emerges. Our findings can guide the design of future GWAS, in particular those utilising the rich multivariate data becoming available from large-scale biobanks such as the UK Biobank, German National Cohort and US Biobank.

We provide a web application (www.MultiTraitGWAS.kcl.ac.uk) that can reproduce all results in this paper, offering flexibility in output and providing interpolated results. A built-in tool generates simple multivariate genetic data sets instantaneously, while a downloadable command-line program can be used to simulate larger, more complex multivariate data and to extend our comparison study with novel methods or under different parameter settings. Finally, our web application acts as a power calculator for multivariate GWAS, which should aid with method selection given available data, and in budgeting proposed studies. Our simulation framework and associated software tool can help to guide the future development and direction of multivariate methodology in genetic epidemiology.

## Results

### Multivariate simulation framework

**Figure 1:**
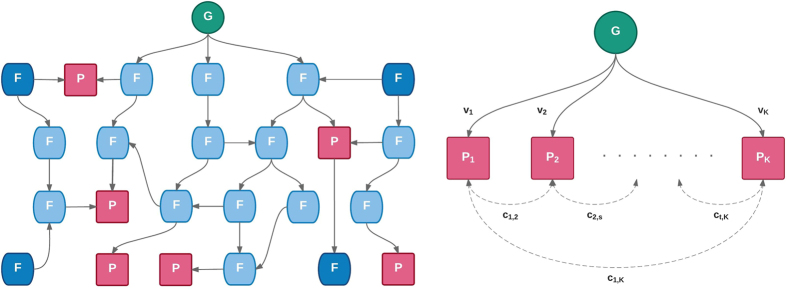
Modelling of multivariate biological network. **(a)** A biological network illustrating a genetic variant (G) influencing a set of biological entities, such as enzymes, metabolites and disease outcomes. Most are unmeasured internal (light blue) or external (dark blue) factors (F), but a subset corresponds to measured phenotypes to be tested (P). **(b)** With no loss in generality, observed phenotype data from a biological network such as that represented in (a) (assuming no indirect genetic effects on observed phenotypes via other observed phenotypes) can be depicted and parameterised by *v* and *c* as shown. Values of *v* and *c* differ from their marginal values when observed risk factors are controlled for.

We construct a simulation framework to model the multivariate network that exists between a single nucleotide polymorphism (SNP) and *K* observed phenotypes, with internal and external risk factors and confounders. Figure 1a illustrates such a network, while Fig. 1b shows how this network can be collapsed with no loss in generality in the context of multi-trait GWAS, resulting in two sets of modelling parameters:


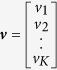



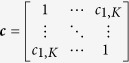


where *v* is the genetic effect vector of the variance in each of the *K* phenotypes explained by the genetic variant, and ***c*** models the phenotypic correlation matrix such that ***c***_s,t_ is the approximate (see below) correlation (Pearson’s correlation coefficient, *r*) between phenotype *s* and phenotype *t*, while the variance of each trait is 1.

The SNP genotypes ***G**_i_* ∈{0, 1, 2} are generated according to Hardy-Weinberg Equilibrium (HWE) in the proportions {*p*^2^, 2*pq, q*^2^} where *p* is the major allele frequency and *q* is the minor allele frequency, with ***q*** = 1−*p*. The genotype-phenotype data are simulated according to the model:


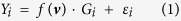


where *Y*_*i*_ = {*Y*_*i,*1_,…, *Y*_*i,K*_} denotes the phenotype data corresponding to *K* phenotypes for an individual *i, f(**v***) denotes the regression coefficient corresponding to *v* phenotypic variance in *Y*_*i*_ explained by the SNP genotypes *G*_*i*_, and ***ε***_*I*_ is the residual variance drawn from the multivariate normal distribution ***N***(0, ***c***) (thus ***c***is not exactly the same as the phenotypic correlation matrix but given small genotype effect sizes is approximately equivalent; henceforth we describe ***c***as the phenotypic correlation matrix). The regression coefficient *f(**v***) is determined according to the following equation, under the assumption of additive genetic effects:


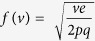


where *ve* is the transformed phenotypic variance explained by the SNP, determined by:


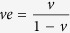


So, for example, to model a SNP that explains 0.5% phenotypic variance, then *ve* = ^0.005^/_0.995_ = 0.005025 since the residual error variance is equal to 1. While this, our main data-generating model, does not consider indirect effects of genetic variants on tested traits via other tested traits, nor case/control phenotype data, we also simulate (see Methods) and investigate these.

By varying the values that *v* and *c* take, genotype-phenotype data consistent with almost any underlying biological network (Fig. 1a) and set of observed phenotypes can be generated. However, since there are infinite values that *v* and *c* can take, a systematic search through the parameter space is required. Our simulation framework aims to capture as much of the parameter space as possible via four modelling scenarios: (S1) *v* and *c* are varied in a structured way, (S2) *v* and *c* sampled from uniform distributions, (S3) *v* and *c* reflect each other, (S4) *v* and *c* informed by real data. Table 1 provides a summary of the simulation scenarios implemented in our simulation framework.

While our simulation framework and associated software will be useful for multivariate methodology development and applications across genetic epidemiology, we exploit it here to perform a comprehensive comparison study of multi-trait GWAS methods.

### Multi-trait GWAS method comparison study

**Figure 2:**
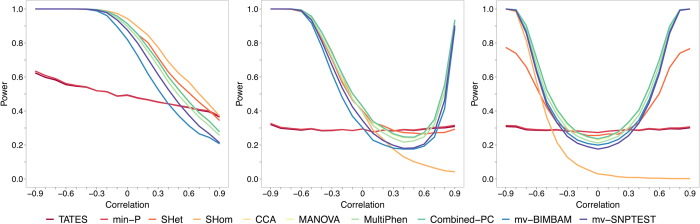
Power of methods under simulation of scenario 1 with two traits. **(a)** The genetic variant explains 0.5% variance in two traits (*v*_1_). **(b)** The genetic variant explains 0.5% variance in one trait and 0.1% in the other (*v*_2_). **(c)** The genetic variant explains 0.5% variance in one trait and has no effect on the other (*v*_3_).

In this comparison study we compare single-SNP multi-trait methods, both those that use individual-level genotype-phenotype data: MANOVA, CCA^12^ (mv-PLINK), Combined-PC^13^, MultiPhen^9^, mv-BIMBAM^14^ and mv-SNPTEST^15^, and those that exploit GWAS summary statistics: min-*P*^9^, TATES^10^, S_Hom_^11^, and S_Het_^11^. These methods cover several approaches to testing the association of genetic variants with multiple phenotypes, including multiple linear regression techniques^12^,^13^, a reversed (ordinal) regression with SNP as outcome^9^, simple^9^ and complex^10^ adjustments of summary statistic results, across trait meta-analysis techniques^11^ and Bayesian methods^14^,^15^ (see Methods). Supplementary Table 1 provides a summary of the methods compared in this study. The simulation framework provides a thorough and consistent platform on which to compare these different methods. We illustrate power across the full range of phenotypic correlations (e.g. Fig. 2), for up to 48 phenotypes, which we believe represents the clearest way to expose differences in method performance.

### S1: Structured genetic effects and phenotypic correlations

In this scenario the genetic effects, *v*, and phenotypic correlations, *c*, are varied in a structured way. First we consider a case with only two phenotypes and three genetic effect vectors:


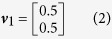



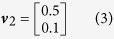



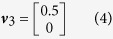


such that *v*_2_ corresponds to a SNP that explains 0.5% variance in trait 1 and 0.1% variance in trait 2. The phenotypic correlations are varied between *r* = −0.9 and *r* = 0.9 in increments of 0.1. Data corresponding to 5,000 individuals are simulated according to Equation 1 under each of the three effect vectors across the range of correlations. 10,000 such replicates of genotype-phenotype data are simulated and statistical power is measured as the proportion of results with a multivariate association *P*-value <5×10^−8^ or a log_10_ Bayes factor >6 for the Bayesian methods (see Methods). Figure 2 shows the statistical power of the 10 multivariate methods when applied to these data.

Figure 2 shows that the methods fall in to one of two distinct groups in terms of their power curves, except for S_Hom_, which has a different pattern in Fig. 2b and Fig. 2c (see below). The min-*P* and TATES methods – which have almost identical power – have lower power across much of the parameter space. In Fig. 2a, we observe a decrease in power for min*-P* and TATES across the correlation range. Both methods perform univariate tests for association with each trait before correcting the smallest *P*-value to account for the number of tests performed (see Methods). When the traits are highly positively correlated the variability in the univariate *P*-values is small, resulting in similar *P*-values for each test. In contrast, when the traits are uncorrelated the two tests are independent and the variability in the univariate *P*-values is greater, increasing the probability that one is small. For negative phenotypic correlations, this variability is even greater, resulting in higher power. However, in Fig. 2b and Fig. 2c, since the genetic effect on the second trait is very small or zero, then the minimum *P*-value will almost certainly derive from the SNP with large effect and is thus invariant to the phenotypic correlation.

The group of methods that follow a different pattern gain power via reducing the residual variance in analysing the traits jointly. When the genetic variant affects both traits equally and the traits are highly correlated (Fig. 2a), these methods lose power due to the limited additional residual variance explained for the same degrees of freedom penalty. When only one trait is affected by the genetic variant and the phenotypes are uncorrelated (Fig. 2c) then there is no gain in residual variance explained by including the unaffected trait. The methods gain power when there is discordance between the genetic effects and the phenotypic correlations, due to the potential increase in explained residual variance^9^. Figure 2c shows that while the S_Het_ method follows the same pattern as the other methods in this group, it has lower power for high positive and negative correlations. This is due to the trait sub-setting procedure of S_Het_ (see Methods), which incurs a relatively strong multiple testing penalty. However, as a summary statistic method S_Het_ can be applied to large publicly available GWAS results and thus may have a substantial boost in power for certain traits.

In Fig. 2 the S_Hom_ method, which performs a meta-analysis on the traits, generally performs best in pleiotropic scenarios. In Fig. 2c only one trait is affected by the genetic variant and when the traits are uncorrelated there is no gain in power by including the unaffected trait; S_Hom_ loses power over min-*P* and TATES here due to it relating to the average rather than maximum association. For highly positively correlated traits, the addition of the unaffected trait in the meta-analysis reduces the average effect size and thus power, but for highly negatively correlated traits the individual effect sizes are augmented, leading to increased power. While a standard meta-analysis would produce a different power curve to that of S_Hom_ in this scenario, the latter explicitly adjusts for the trait correlations, which provides a well-behaved statistic under the null. The same explanation applies to Fig. 2b, although here the loss in power for S_Hom_ is less pronounced due to the small genetic effect on the second trait.

While the number of qualitatively different genetic effect vectors is only three for two phenotypes (equal effects, different effects, one effect and one with no effect), the number increases exponentially as more traits are considered. In this scenario and scenario S3 below, we consider 2, 4, 8, 20 and 48 phenotypes and for those with 4 or more we apply 10 genetic effect vectors, defined in Table 2. In scenarios S2 and S4a we consider 2, 4 and 8 phenotypes (see below).

**Figure 3:**
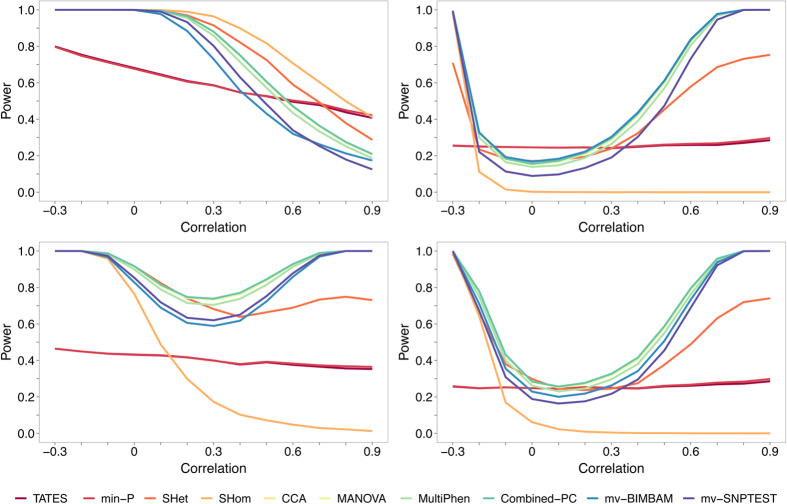
Power of methods under simulation of scenario 1 with four traits. Power comparisons from simulations of scenario 1 (S1), based on **(a)** *v*_1_, **(b)** *v*_4_, **(c)** *v*_8_ and **(d)** *v*_10_ (see Table 2) applied to data on 4 phenotypes. For all scenario 1 (S1) results the correlations between all phenotypes are the same. Correlations <−0.3 are not possible across 4 phenotypes, hence the truncation in these – and subsequent - results across the correlation range. Full results for scenario 1 (S1) are shown in Supplementary Figures 1–4.

Figure 3 illustrates the results corresponding to *v*_1_, *v*_4_, *v*_8_, and *v*_10_ in relation to 4 phenotypes, and Supplementary Figures 1–4 show the results for the remaining genetic effect vectors for 4 phenotypes and for all 10 genetic effect vectors (Table 2) in relation to 8, 20 and 48 phenotypes. A clear pattern emerges across the results. The individual-level methods form a ‘leading group’ in terms of power across much of the parameter space, in contrast to the lower performing pair of methods min-*P* and TATES, while S_Hom_ and S_Het_ tend towards this leading group the more pleiotropic the scenario (e.g. *v*_1_). S_Het_ has markedly higher power than the other summary statistic methods in most scenarios and often similar to the individual-level data methods. In the most pleiotropic scenario, *v*_1_, S_Hom_ performs best and min-*P* and TATES outperform the individual-level methods under high, positive phenotypic correlations (e.g. Fig. 3a); otherwise S_Hom_ performs poorly. These differences in power between the methods increase with a greater number of phenotypes (see Supplementary Figs 1–4).

Supplementary Figure 5 shows the behaviour of the methods under the null hypothesis of no direct genetic effects on all phenotypes. While the methods generally perform as expected under the null, there is mild inflation for min-*P* and TATES under high phenotypic correlations for ≤ 8 phenotypes and for MultiPhen for 48 phenotypes, and strong deflation for min-*P*, TATES and S_Hom_ for 48 phenotypes. Therefore, use of these methods in these scenarios should either be avoided or else their statistics should be adjusted so that the error rates are controlled.

Our main data-generating model (Equation 1) does not consider indirect genetic effects, whereby the genetic variant has an effect on one of the tested phenotypes via its effect on another (a *downstream* or *mediated* effect). Here we perform simulations that model such an effect across two phenotypes (see Methods). We simulate the genetic variant as explaining 0.5%variance in the first phenotype, and the first phenotype explaining 1%, 5%, 10% and 20% variance in the second phenotype. The results from these simulations (Supplementary Fig. 6) closely reflect those above in which there is a strong direct effect on one phenotype and no or a small direct effect on the other. Given that a genetic effect on a trait will be sharply attenuated when it is only exerted via its effect on another tested trait, then unless the phenotypes are highly similar we expect our results on direct effects to capture the vast majority of those relating to indirect effects as well. One exception may be the results of the Combined-PC test, which depart from the other individual-level data methods over some of the parameter space when the first trait has a very large effect on the second (see Supplementary Fig. 6d); large differences in results between the Combined-PC test and the other individual-level data methods on real data may thus indicate the presence of an indirect effect, which may inspire a test to distinguish direct and indirect effects. Supplementary Figure 7 indicates that the behaviour of the methods under the null in the context of downstream effects reflects that for direct effects.

To examine the relative power of the methods when applied to case/control data, we simulated varying genetic effects on a pair of case/control phenotypes and on the combination of a case/control phenotype and a quantitative trait (see Methods). Supplementary Figure 8 shows the results for the 7 multivariate methods that can be applied to case/control data. The results show a similar pattern to those on quantitative traits only, where we observe two patterns for the power of the methods and departure from these patterns for the S_Hom_ method in non-pleiotropic scenarios. Min-*P* and TATES appear to perform relatively better when applied to case/control data in general but worse when the genetic variant affects both phenotypes equally, while S_Hom_ performs poorly when there is an effect on only one of the phenotypes, as expected.

### S2: Genetic effects and phenotypic correlations sampled uniformly

In contrast to the structure of the S1 simulations, here we simulate data with genetic effects and phenotypic correlations sampled from uniform distributions. Data are re-simulated if phenotype correlation matrices are not positive-definite and genetic effect sizes are bounded between 0% and 0.5% phenotypic variance explained, but otherwise each of the 10,000 genotype-phenotype simulated datasets relate to a random combination of genetic effects and phenotypic correlations. Supplementary Figure 9 indicates that the individual-level data methods, as well as S_Het_ and S_Hom_, have almost identical power and distinctly higher than that of min-*P* and TATES, with the difference larger for a greater number of traits. These results are in broad agreement with those of scenario 1 (S1), where this leading group of methods generally outperforms min-*P* and TATES.

### S3: Genetic effects that reflect phenotypic correlations

Since genetic variants mostly explain <1% of phenotypic variance^26^,^27^, their effects on a set of phenotypes do not induce phenotypic correlations reflecting their relative sizes. However, it may be likely that genetic effects are on average more reflective of the corresponding phenotypic correlations than not. Here we simulate data such that the phenotypic correlations reflect the relative sizes of the genetic effects. Phenotypic correlations are chosen to reflect the genetic effect vectors described in scenario 1 (S1) (three effect vectors for 2 traits, and 10 for ≥4 traits) in the following way: if the variant has equal effects on a pair of traits then the corresponding pairwise phenotypic correlation is set to be 0.6, for different effect sizes the pairwise correlation is 0.2, and if the variant affects only one of the phenotypes then their pairwise correlation is set as 0.05. As in scenario 1 (S1), the power of the methods will be a function of the phenotypic correlations modelled, the impact of which can be further explored using our simulation tool.

**Figure 4:**
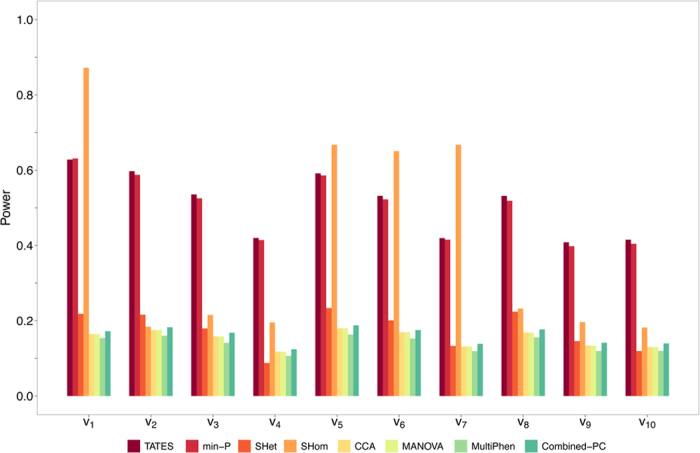
Power of methods under simulation of scenario 3 with 20 traits. Power comparisons for all simulations of scenario 3 (S3) involving 20 phenotypes. In this scenario the phenotypic correlations are chosen to reflect the relative genetic effect sizes defined by the 10 genetic effect vectors (see Table 2 and description under S3 sub-heading of main text). mv-BIMBAM was not computationally feasible, and mv-SNPTEST not hard-coded, for 20 or more phenotypes and so were excluded here. All other results for scenario 3 (S3) are shown in Supplementary Figure 10.

While the results for two phenotypes are mostly similar across all methods (see Supplementary Fig. 10a), the summary statistic methods generally outperform the individual-level data methods more as the number of traits increases. The results of S_Hom_, however, are sensitive to the genetic effect vector, being the best or worst performing summary statistic method depending on the genetic effects, while the power of S_Het_ and the individual-level data methods is greatly reduced for 20 and 48 traits. These results are in broad agreement with those of scenario 1 (S1) in which the genetic effects and phenotypic correlations are concordant. Figure 4 illustrates the results for 20 phenotypes and Supplementary Figure 10 shows all other results for this scenario.

### S4: Real data informed simulations

The final simulation scenario exploits real data from published GWAS results to simulate realistic values for the genetic effect and phenotype correlation parameters. This scenario is in two parts: (a) real data informed phenotype correlations, and (b) real data informed genetic effects and phenotype correlations.

- *Real data informed phenotype correlations.* Here we draw the phenotypic correlations from a mixture of Gaussian distributions modelled on real phenotypic correlations corresponding to 16 phenotypes from the Northern Finland Birth Cohort 1966 (NFBC1966) (see Methods and Supplementary Fig. 11). Genotype-phenotype data are generated as in scenario 1 (S1) but by sampling the pairwise phenotypic correlations from this fitted density, discarding sampled non-positive-definite correlation matrices. Supplementary Figure 12 reveals that the individual-level methods and S_Het_ have markedly higher power than min-*P* and TATES for the majority of the genetic effect vectors. The performance of S_Hom_ is, again, highly dependent on the genetic effect vector, with greatest performance under pleiotropic effects. The results from this scenario are similar to those of scenario 2 (S2); in both cases the genetic effect vectors are independent of the phenotypic correlations, which optimises the statistical power of the individual-level data methods and S_Het_.
- *Real data informed genetic effects and phenotype correlations* Here we sample genetic effect sizes directly from reported genotype-phenotype associations from publicly released GWAS on 12 continuous phenotypes: height^28^, BMI^29^, systolic blood pressure^2^, diastolic blood pressure^2^, triglycerides^30^, HDL^30^, LDL^30^, total cholesterol^30^, fasting-glucose^31^, fasting-insulin^31^, HOMA-B^31^ and HOMA-IR^31^. We compiled a list of all SNPs with a reported genome-wide significant association in the largest available GWAS on each trait, and recorded the corresponding genetic effect size for each SNP across all 12 traits. This provided 237 SNPs and hence 237 genetic effect size vectors. We then simulated 10,000 replicates of genotype-phenotype data corresponding to 5,000 individuals by sampling from these genetic effect vectors and directly (not from the density of Supplementary Fig. 11) from phenotypic correlations estimated in the NFBC1966 data on those traits (see Methods). As well as a comparison based on all 12 phenotypes, we repeated this scenario for 2, 4 and 8 phenotypes by iterating through all ^12^*C*_*K*_ combinations of the 12 phenotypes, and forming the corresponding genetic effect vectors and phenotype correlation matrices to simulate data.

**Figure 5:**
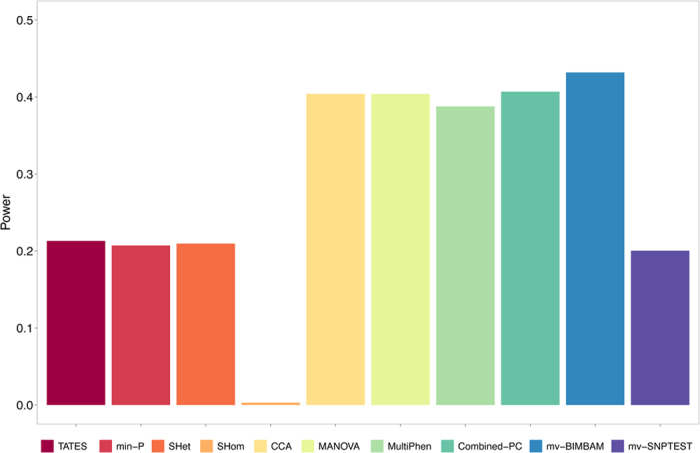
Power of methods in real data informed simulations with 12 traits. Power comparisons for the real data informed simulations of scenario 4b (S4b) involving 12 phenotypes. In this scenario both genetic effects and corresponding phenotypic correlations are drawn directly from real data on the same set of traits. All other results for this scenario are shown in Supplementary Figure 13.

These results, shown in Figure 5 for 12 traits and Supplementary Figure 13 for 2, 4 and 8 traits, may provide the most informative overall comparison of method performance given their basis on real combinations of effects and correlations. For 2 phenotypes, the individual-level methods substantially outperform min-*P*, TATES and S_Hom_, while S_Het_ has similar power (see Supplementary Fig. 13). As the number of phenotypes increases the power of the summary statistic methods decreases, with S_Het_ and S_Hom_ having the most dramatic decreases in power. For 12 phenotypes, S_Het_ has similar power to min-*P* and TATES, while S_Hom_ performs particularly poorly in this scenario. From the results on 12 phenotypes we would expect the individual-level data methods to yield approximately twice the discovery of genetic variants than the summary statistic methods when applied to real data studies of the same sample size. According to the results of our other simulations (e.g. Supplementary Figs 9 and 12), this higher power for the individual-level methods suggests that the genetic effects on correlated traits may often only weakly reflect the degree of phenotypic correlation. However, for studies that can utilise much larger resources of summary statistic data than individual-level data, applying a summary statistic method may optimise statistical power. To explore this, we simulated genotype-phenotype data relating to 10,000 individuals and performed simulations using the best performing summary statistic method, S_Het_, to evaluate the potential power gains. The results, shown in Supplementary Table 2, indicate that S_Het_ has substantially higher power than the individual-level data methods at this increased sample size, although its advantage reduces with more traits. The expected power of the methods in studies exploiting individual-level or summary statistic data of different sizes can be further estimated using our web application and software program.

## Discussion

We have presented a simulation framework for generating data relating genetic variants with multiple phenotypes. The framework incorporates a range of simulation scenarios to explore the vast model space relevant to multivariate genetic data. While we have exploited the framework for a multi-trait GWAS methods comparison here, it should have wide application across genetic epidemiology and with minor modifications could be exploited to model any network of correlated variables for which a subset are influenced by a common factor. We have implemented our simulation framework as a web application and open-source software program with flexible user options, so that others can extend our study to incorporate different modelling scenarios and benchmark additional multivariate GWAS methods.

Development of multi-trait GWAS methodology has been an active area of research in recent years^7^,^8^,^9^,^10^,^11^,^12^,^13^,^14^,^15^,^16^,^17^,^18^,^19^,^20^. However, publications introducing new methods are highly inconsistent in their evaluation of method performance, obscuring their relative merit. Here we have provided a consistent platform for benchmarking methods of sufficient rigor to expose their differences and similarities, and to demystify user choice.

In the structured simulations of scenario 1 (S1), the individual-level data methods and the meta-analysis approaches of S_Het_ and S_Hom_ mostly outperform the univariate approaches of min-*P* and TATES. However, such a structured search of the model space may lead to testing unrealistic data, such as a genetic variant affecting only 12 of 48 traits, whose pairwise correlations are all 0.9. Therefore, some observed power differences may apply only to particular groups of traits or in settings outside genetic epidemiology. When genetic effects and phenotypic correlations are sampled from uniform distributions (S2), S_Het_, S_Hom_ and the individual-level data methods show markedly higher power than TATES and min-*P*. This is consistent with a general tendency for the individual-level data methods to have greatest relative power when the genetic effects and phenotypic correlations are discordant. This is further supported by the results from scenario 3 (S3), where the genetic effects and phenotypic correlations reflect each other. Here the summary statistic methods tend to perform best, especially S_Hom_ in the scenarios that are most pleiotropic. In the final scenario (S4b), genetic effects and phenotypic correlations are based on real data, and in the results relating to 12 traits the individual-level methods provide twice the discovery of genetic variants over the summary statistic methods.

Overall our results suggest that for a given sample size, the individual-level methods tested here are likely to optimise the discovery of genotype-phenotype associations. However, it should be noted that a summary statistic method with the same underlying assumptions as an individual-level method could be developed in the future, and thus reduce the gap in power between the two types of method. It should also be noted that by using fixed thresholds of *P* < 5 × 10^−8^ and log10 Bayes factor >6 we have not ensured equal type 1 error rates of the methods throughout comparisons; therefore, the fine-scale power differences between the methods estimated here, in particular in comparing the Frequentist and Bayesian methods, should not be strongly interpreted. The choice of which individual-level method to use depends, in part, on the computational feasibility for the number of traits being analysed (see Supplementary Table 3). For example, mv-BIMBAM has highest power in scenario 4b (S4b) on 12 traits, but becomes computationally infeasible for a large number of traits (≥10). Other individual-level methods, in particular CCA, are preferable for a larger number of traits in terms of computation time (Supplementary Table 3). The mv-BIMBAM method also provides additional interpretation by assigning probabilities to the combinations of direct, indirect and no effect of a SNP on the traits analysed, which provides insights into the genetic aetiology underlying multiple traits. If summary statistics are available on a sample that is markedly larger in size than that of available individual-level data then it is highly likely that applying S_Het_, in particular, will yield greatest power (Supplementary Table 2). S_Hom_ is the best choice if the objective is to identify genetic variants with highly pleiotropic effects across all phenotypes under study.

In addition to providing a comprehensive guide to method choice in multi-trait GWAS, the extensive array of scenarios considered here expose several issues relating to the methods, not established in previous publications: (i) despite the sophisticated adjustment of univariate *P*-values performed by TATES, its power is approximately equivalent to simply adjusting the minimum *P*-value of the univariate tests by the effective number of independent tests (min-*P*); (ii) while the Combined-PC method shows almost equivalent power to the other individual-level data methods throughout, it has a marked departure in power for indirect genetic effects on the tested traits - this could provide a simple method for distinguishing direct and indirect effects; (iii) in many scenarios, S_Het_ and S_Hom_ have similar power to the individual-level data methods, demonstrating the potential for summary statistics to provide as much information as individual-level multivariate data; (iv) most multi-trait methods are not optimised for identifying pleiotropic variants^32^, despite common reference to pleiotropy in publications that apply them; and (v) S_Hom_, which is tailored to detect pleiotropic variants, performs poorly in the real data informed simulations relating to 12 traits, suggesting that tests for pleiotropic variants may not produce novel findings unless applied to phenotypes that have prior knowledge of shared genetic aetiology.

While we have assessed the performance of many of the leading multi-trait GWAS methods across a range of different scenarios, we acknowledge that there are many scenarios not considered here. For example, we did not consider the situation where only one of many traits is affected by the genetic variant; in this setting we may expect min-*P* and TATES to perform well, despite our overall findings that these methods appear to have sub-optimal power. However, such simulations are easily performed using our simulation software, which implements the scenarios considered here and allows flexibility in user choice over parameters such as the number of traits, genetic effects and phenotypic correlations in order to simulate new scenarios. The results of this highly non-pleiotropic scenario, where we simulate 48 traits with only one causal association, are presented in Supplementary Figure 14. Min-*P* and TATES do indeed perform well in this scenario, although the individual-level methods have greater power for higher phenotypic correlations. We also recognise that there are other multivariate approaches to genetic association studies that have not been considered here, such as linear mixed models^8^, generalised estimating equations^19^ and adaptive testing^20^, as well as multi-SNP, multi-trait approaches^7^,^8^,^20^. It is possible that under some assumptions these methods are preferred over those considered here, though our results suggest that the power of most multivariate methods converge to some optimal value for a large part of the model space. Additional methods can be easily incorporated into our simulation software, allowing further methods to be benchmarked under a wide range of simulation settings.

Multivariate genetic analyses are likely to expand dramatically in future as an increasing number of GWAS results are released publicly and as individual-level multivariate panels are compiled by population-wide biobank studies. This makes our study extremely timely, and designing studies guided by its findings should lead to greater discovery of genuine associations. This could be especially significant for underpowered but extremely important phenotypes, such as depression^33^,^34^, for which few genetic associations have been discovered. Multivariate methods may leverage the power of GWAS on such phenotypes, providing vital targets for drug development in diseases and disorders with few biological leads. In addition to the direct benefits of increasing the number of known genetic associations for any phenotype, without cost, this will also produce higher-powered downstream analyses, such as pathway analyses and polygenic risk scoring.

While our results provide a present snapshot of multi-trait GWAS method performance, our simulation framework offers a consistent platform from which future methods can be easily benchmarked via our web application and open-source software program. This should save researcher time and avoid repetition by guiding the development, application and publication of only those methods demonstrated as outperforming the alternatives. We believe that this study highlights the importance of systematic and comprehensive comparisons of competing methods of analysis, easily reproduced and extended via open-source software.

## Methods

### Multi-trait GWAS methods

The 10 methods used in the comparison study are briefly described below, and are summarised in Supplementary Table 1.

#### min-P

This test was proposed in O’Reilly *et al*.^9^ as a way of comparing MultiPhen to a simple multi-trait approach that exploits only existing GWAS univariate summary statistics. First, the minimum *P*-value from the group of *K P*-values corresponding to the *K* phenotypes under study is recorded for every SNP, using the published univariate GWAS results. Next the effective number of independent tests represented by the results on the *K* phenotypes is estimated using the correlation matrix of the phenotypes according to Nyholt^35^, and then the recorded minimum *P*-value is adjusted according to this number of tests in a standard Šidák correction^36^.

#### TATES

This test^10^ is similar to that of min-*P* but performs a more sophisticated correction for multiple testing across the different phenotype results. Here the results are ranked according to *P*-value and then the extended SIMES procedure of Li *et al*.^37^ is performed – on multiple traits rather than variants – by progressively re-ordering the minimum *P*-value according to a scaling that is a function of the effective number of independent *P*-values.

### S_Hom_

This test^11^ combines Wald test statistics from univariate GWAS summary statistics relating to a SNP across both multiple cohorts and multiple phenotypes in a meta-analysis. Heterogeneity in effect size and statistical power across cohorts is accounted for, as is the correlation among the test statistics, while the overall test statistic has optimal power when the genetic effect is homogeneous across traits and cohorts. Of the 10 tests considered here, S_Hom_ is that which can be most considered a ‘test for pleiotropy’, being equivalent to a meta-analysis of effect sizes across traits and cohorts with optimal power under fixed effects.

#### S_Het_

This test^11^ is derived from S_Hom_ but is designed to detect genetic variants that only affect a subset of the total number of traits under study. Only those traits with a corresponding Wald test statistic above some threshold are included in the calculation of a statistic equivalent to that of S_Hom_. This is then recalculated across the range of possible threshold values with the maximum value obtained being the test statistic S_Het_. Since this S_Het_ statistic does not follow a standard theoretical distribution, *P*-values are computed via simulation of a Gamma distribution.

#### MANOVA

The standard Multivariate Analysis of Variance statistical test, which is the multivariate extension of ANOVA, and equivalent to a reversed multiple linear regression with genetic variant as outcome^9^.

#### CCA (mv-PLINK)

Canonical Correlation Analysis refers to a statistical procedure for identifying and testing the association of linear combinations of two sets of variables that maximise their correlation. While this approach could theoretically be applied to test for association between multiple genetic variants and traits jointly, in the context of multi-trait, single-SNP analyses, as incorporated into PLINK by Ferreira and Purcell^12^, this test is equivalent to a reversed multiple linear regression with a single genetic variant as outcome^9^. This method is equivalent to MANOVA, and has been shown to be approximately equivalent to MultiPhen for common SNPs^9^.

#### Combined-PC

This test^13^ performs a principal components analysis (PCA) on the phenotype data. Separate simple linear regressions are performed each with a different PC as outcome and SNP as predictor, and then the chi-squared statistics corresponding to the SNP-PC association from each regression are summed. Since the PCs are orthogonal to each other these tests are independent and their results can thus be summed in this way. Given small genetic effect sizes, the simple linear regressions of PC on SNP are approximately equivalent to reverse regressions of SNP on PC; the sum of these individual regressions is then equivalent to a multiple linear regression with PC predictors. Since all PCs are included as predictors, this is equivalent to a multiple linear regression with phenotype predictors and SNP as outcome, and thus overall the Combined-PC method is approximately equivalent to CCA (mv-PLINK).

#### MultiPhen

This test^9^ performs a ‘reversed regression’, with multiple phenotype predictors and genetic variant as outcome. Since genotypes of SNPs (and other genetic variants) correspond to ordinal data, an ordinal regression is performed here. This test has been shown to be equivalent to MANOVA and CCA for common SNPs^9^.

#### mv-BIMBAM

This test^14^ performs Bayesian multivariate regression by partitioning the phenotypes according to the effect of the genetic variant on them: direct, indirect or no effect. Statistical power is assessed using a log_10_ Bayes factor threshold of 6, following Stephens and Balding^38^ and Shim *et al*.^39^.

#### mv-SNPTEST

This test^15^ performs Bayesian multivariate regression using an inverse Wishart distribution and matrix normal priors. The fit of the full model is compared to that of the null model, and a log_10_ Bayes factor quantifies the association between SNP and phenotypes. Statistical power is assessed using a log_10_ Bayes factor threshold of 6 (ref. 38).

### Modelling indirect effects

We simulate and consider indirect genetic effects for scenario 1 (S1). We model an indirect genetic effect from a SNP *G*_*i*_ to a phenotype *Y*_*i*,2_ by simulating a direct effect on a phenotype *Y*_*i*,1_ and a direct effect from *Y*_*i*,1_ to *Y*_*i*,2_. Data are generated according to the following equations:


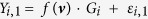



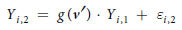


where *f(v*)denotes the regression coefficient corresponding to *v* phenotypic variance in *Y*_*i*,1_ explained by the SNP, *g(v*′) denotes the regression coefficient corresponding to *v* phenotypic variance in *Y*_*i*,2_ explained by *Y*_*i*,1_, and *ε~*N(**0**, ***c***). We only simulate downstream effects for two phenotypes, but the simulations can be easily extended to incorporate more phenotypes, as well as more complex interaction networks.

### Modelling case/control data

We simulate and consider case/control data for scenario 1 (S1). We first simulate quantitative phenotype data as in **Equation 1**, and then apply a liability threshold model of disease^40^ to generate case/control phenotype data according to the prevalence of the disease:


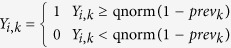


where *prev*_*k*_ is the prevalence of the *k*^th^ phenotype.

### Phenotypic correlations modelled on NFBC1966 data

In scenario 4a (S4a), we fit a mixture Gaussian distribution to the observed NFBC1966 phenotype correlations on 16 metabolic traits:


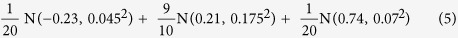


and sample the pairwise phenotypic correlations from this distribution.

### Genetic effects and phenotypic correlations drawn from real data

In scenario 4b (S4b), we use the *β* (SNP effect size) estimates from published GWAS^2^,^28^,^29^,^30^,^31^ to inform the simulation of genetic effects. For a given SNP *G*_*i*_ we take the effect size estimates across *K* phenotypes, say *β*_1_,…,*β*_*K*_, and apply a transformation so that the maximum effect size is 0.5% of phenotypic variance, while maintaining the relative effect sizes. The transformation factor *d*_*i*_ for SNP *G*_*i*_ is defined as follows:


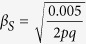


where 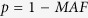 and 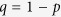, and:


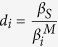


where 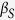 is the beta coefficient that corresponds to the maximum effect size of 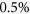 variance explained, and 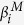 is the maximum beta for a given SNP 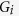
across all *K* phenotypes. The real data obtained beta coefficients for SNP *G*_*i*_ are then multiplied by *d*_*i*_ to generate the beta coefficients used to simulate the phenotype data as in **Equation 1**.

The real data simulations are performed on 12 phenotypes, as well as all possible subsets of 2, 4 and 8 phenotypes from these 12. In each case, we limit the number of SNPs associated with any one phenotype to a maximum of 20, then use this subset of SNPs to derive the betas as above. This aims to prevent bias towards phenotypes with a much larger number of associated SNPs.

## Additional Information

**How to cite this article**: Porter, H. F. and O’Reilly, P. F. Multivariate simulation framework reveals performance of multi-trait GWAS methods. *Sci. Rep.* **7**, 38837; doi: 10.1038/srep38837 (2017).

**Publisher's note:** Springer Nature remains neutral with regard to jurisdictional claims in published maps and institutional affiliations.

## Supplementary Material

Supplementary Information

## Figures and Tables

**Table 1:** Simulation scenarios.

Scenario	Phenotypes	Genetic Effects	Phenotypic Correlations
S1	2, 4, 8, 20 and 48	Fixed genetic effects defined by *v*_1_−*v*_3_ for 2 traits and Table 2 for 4 or more traits	Pairwise phenotypic correlations range between −0.9 and 0.9 in increments of 0.1
S2	2, 4 and 8	Genetic effects sampled uniformly between 0% and 0.5% phenotypic variance explained	Phenotypic correlations sampled uniformly, ensuring the resulting correlation matrix is positive definite
S3	2, 4, 8, 20 and 48	Fixed genetic effects defined by *v*_1_−*v*_3_ for 2 traits and Table 2 for 4 or more traits	Pairwise phenotypic correlations chosen to reflect the genetic effects
S4a	2, 4 and 8	Fixed genetic effects defined by *v*_1_−*v*_3_ for 2 traits and Table 2 for 4 or more traits	Phenotypic correlations sampled from a fitted mixture Gaussian distribution based on NFBC1966 data
S4b	2, 4, 8 and 12	Genetic effects based on univariate GWAS summary statistics	Phenotypic correlations obtained directly from the NFBC1966 on the phenotypes for which the genetic effects were obtained

Summary of the simulation scenarios implemented in the simulation framework and associated software package; full details provided in the **Results** section.

**Table 2:** Genetic effect vectors used for simulations of 4 or more phenotypes.

Genetic effect vector	1^st^ 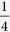 of traits	2^nd^ 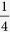 of traits	3^rd^ 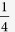 of traits	4^th^ 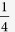 of traits
*v*_1_	0.5	0.5	0.5	0.5
*v*_2_	0.5	0.5	0.5	0
*v*_3_	0.5	0.5	0	0
*v*_4_	0.5	0	0	0
*v*_5_	0.5	0.5	0.5	0.1
*v*_6_	0.5	0.5	0.1	0.1
*v*_7_	0.5	0.1	0.1	0.1
*v*_8_	0.5	0.5	0.1	0
*v*_9_	0.5	0.1	0.1	0
*v*_10_	0.5	0.1	0	0

Description of the 10 genetic effect vectors used in the simulations of scenarios S1, S3 and S4a that simulate a genetic effect on ≥4 phenotypes. These effect vectors are chosen to cover a large proportion of qualitatively different combinations of genetic effects as efficiently as possible via assigning genetic effects to each 1/4 of the traits. For 8 phenotypes, *v*_5_ corresponds to the genetic variant explaining 0.5% variance in 6 of the traits and 0.1% in 2 of the traits, while for 20 phenotypes *v*_8_ corresponds to the genetic variant explaining 0.5% variance in 10 traits, 0.1% variance in 5 traits and having no effect on 5 traits.
