# Supplementary material for: Multivariate simulation framework reveals performance of multi-trait GWAS methods
Source: Sci Rep. 2017 Mar 13;7:38837. doi: 10.1038/srep38837 (PMC5347376; doi:10.1038/srep38837)
Supplement: Supplementary Information [file srep38837-s1.pdf]

ARTICLE

Multivariate simulation framework reveals performance of multi-trait GWAS methods

Heather F Porter[1], Paul F O'Reilly[1,*]

Affiliations:
[1]MRC SGDP Centre, Institute of Psychiatry, Psychology & Neuroscience, King's College London, London, SE5 8AF, UK.

Correspondence:
*paul.oreilly@kcl.ac.uk

Supplementary Information

Summary of the multi-trait GWAS methods compared

Method		Data Type	Information
Univariate	min-*P*[9]	Univariate *P*-values	Adjusts univariate *P*-values in a standard Šidák[36] correction, using the effective number of tests of Nyholt[35]
	TATES[10]	Univariate *P*-values	Adjusts univariate *P*-values using the *P*-value correlation matrix as determined by the phenotypic correlation matrix
Summary statistic	S_{Hom}[11]	Univariate t-values	Performs a meta-analysis across traits weighted by the t-value correlations and the univariate study sample sizes
	S_{Het}[11]	Univariate t-values	Performs the test of S_{Hom} but for subsets of traits at each SNP; subsets are determined by a user specified t-value threshold
Individual-level genotype-phenotype data	MANOVA	Normally distributed	Test equivalent to multiple linear regression with phenotype predictors and genotype outcome
	CCA (mv-PLINK)[12]	Normally distributed	Performs multiple linear regression with phenotype predictors and genotype outcome
	MultiPhen[9]	Ordinal variable	Performs ordinal logistic regression with phenotype predictors and genotype outcome
	Combined-PC[13]	Normally distributed	Performs a test equivalent to multiple linear regression with PC predictors and genotype outcome
	mv-BIMBAM[14]	Normally distributed	Performs Bayesian multivariate regression, sub-setting the traits according to their SNP effect: direct, indirect or no effect
	mv-SNPTEST[15]	Normally distributed	Performs Bayesian multivariate regression using a conjugate prior (Wishart on covariance matrix, matrix normal on the genetic effects)

Supplementary Table 1. Summary of the 10 multi-trait GWAS methods used in the comparison study.

S1: Results for 4, 8, 20 and 48 phenotypes

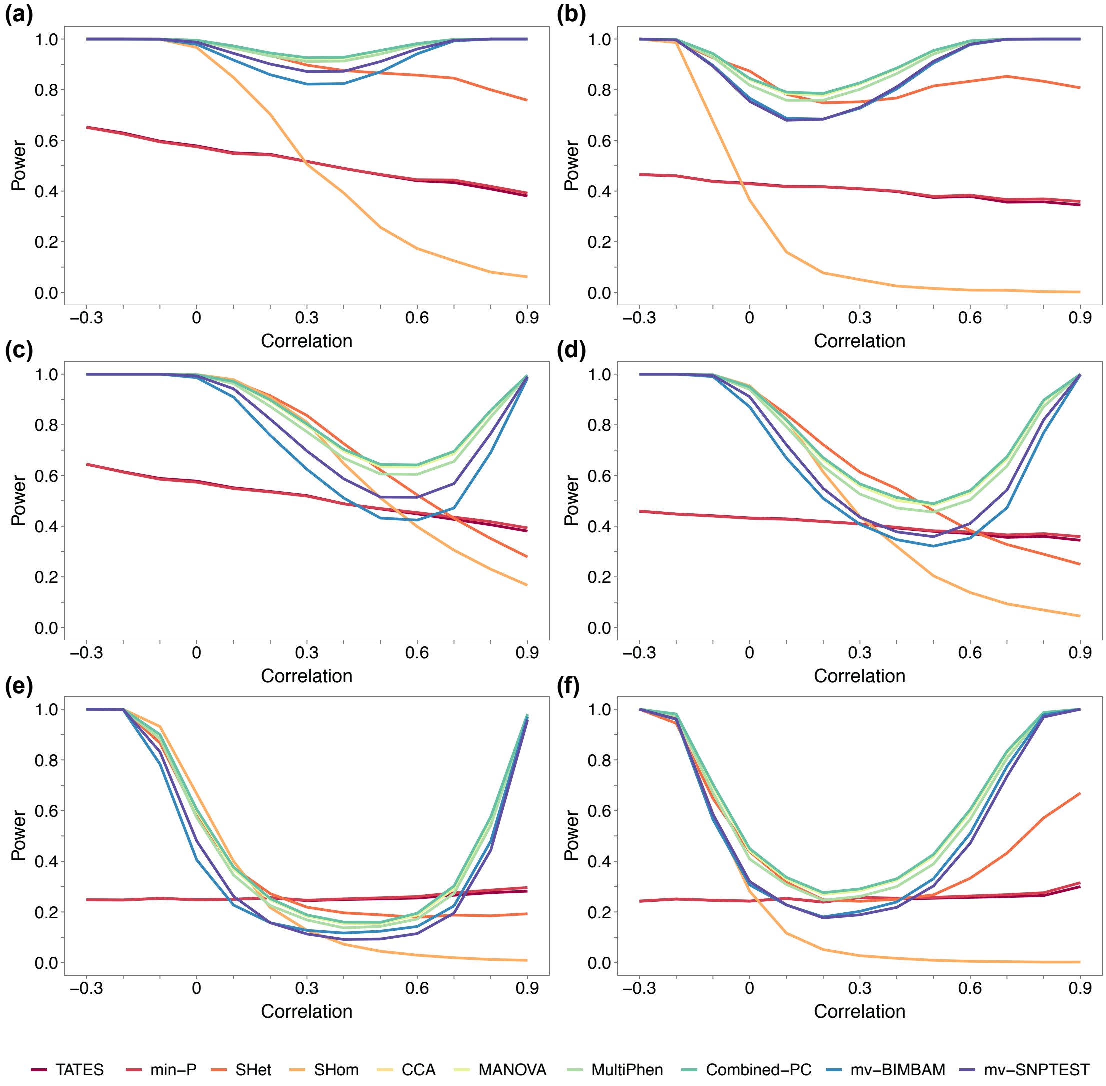

Supplementary Figure 1. Power comparisons from simulations of scenario 1 (S1), based on **(a)** v_2, **(b)** v_3, **(c)** v_5, **(d)** v_6, **(e)** v_7 and **(f)** v_9 (see **Table 2**) applied to data on 4 phenotypes. The pairwise phenotypic correlations are the same for all phenotypes. Correlations < -0.3 are not possible across 4 phenotypes, hence the truncation in these results across the correlation range.

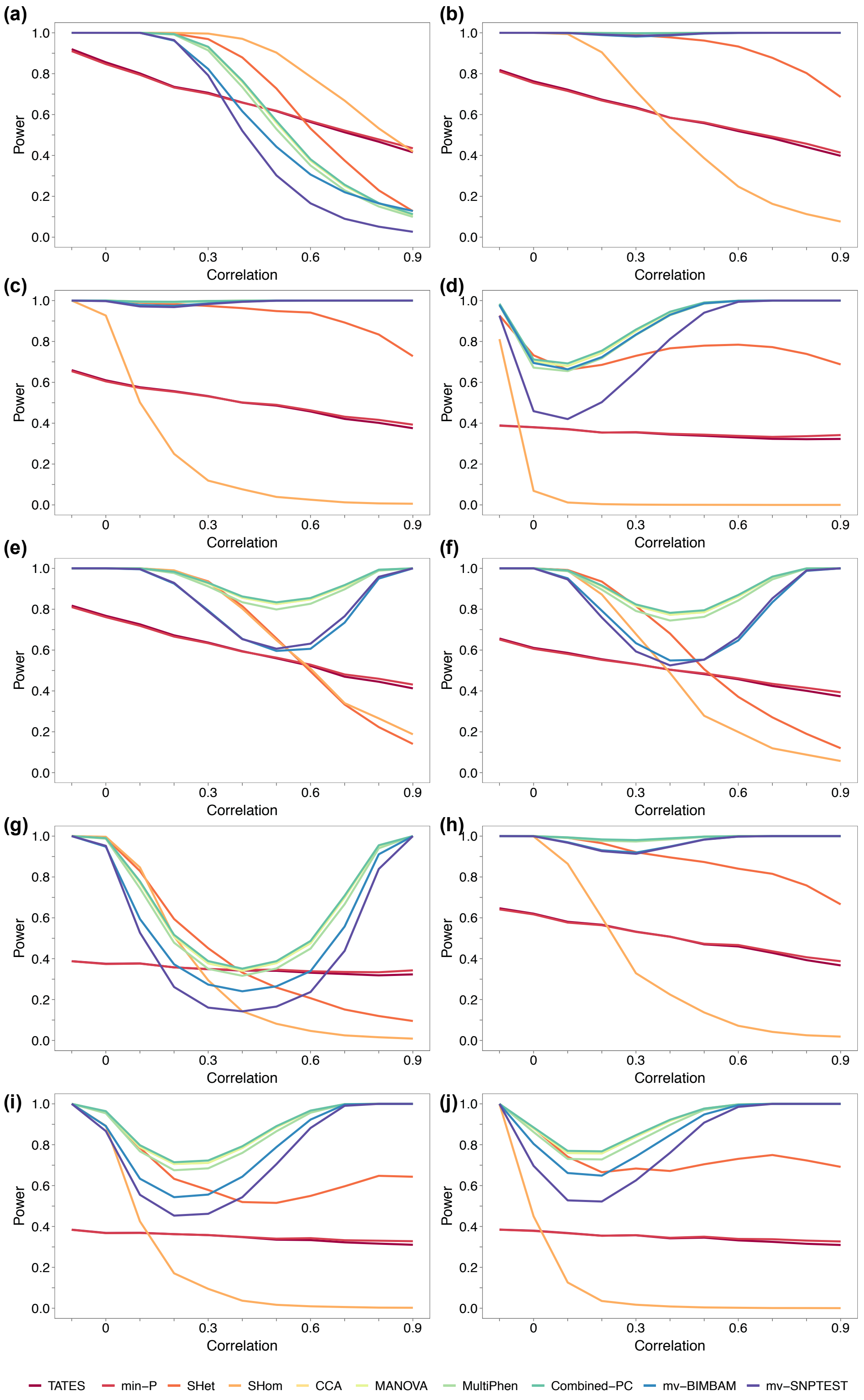

Supplementary Figure 2. Power comparisons from simulations of scenario 1 (S1), based on $v_1 - v_{10}$ (see **Table 2**) applied to data on 8 phenotypes, **(a)** – **(j)** respectively. The pairwise phenotypic correlations are the same for all phenotypes. Correlations < -0.1 are not possible across 8 phenotypes, hence the truncation in these results across the correlation range.

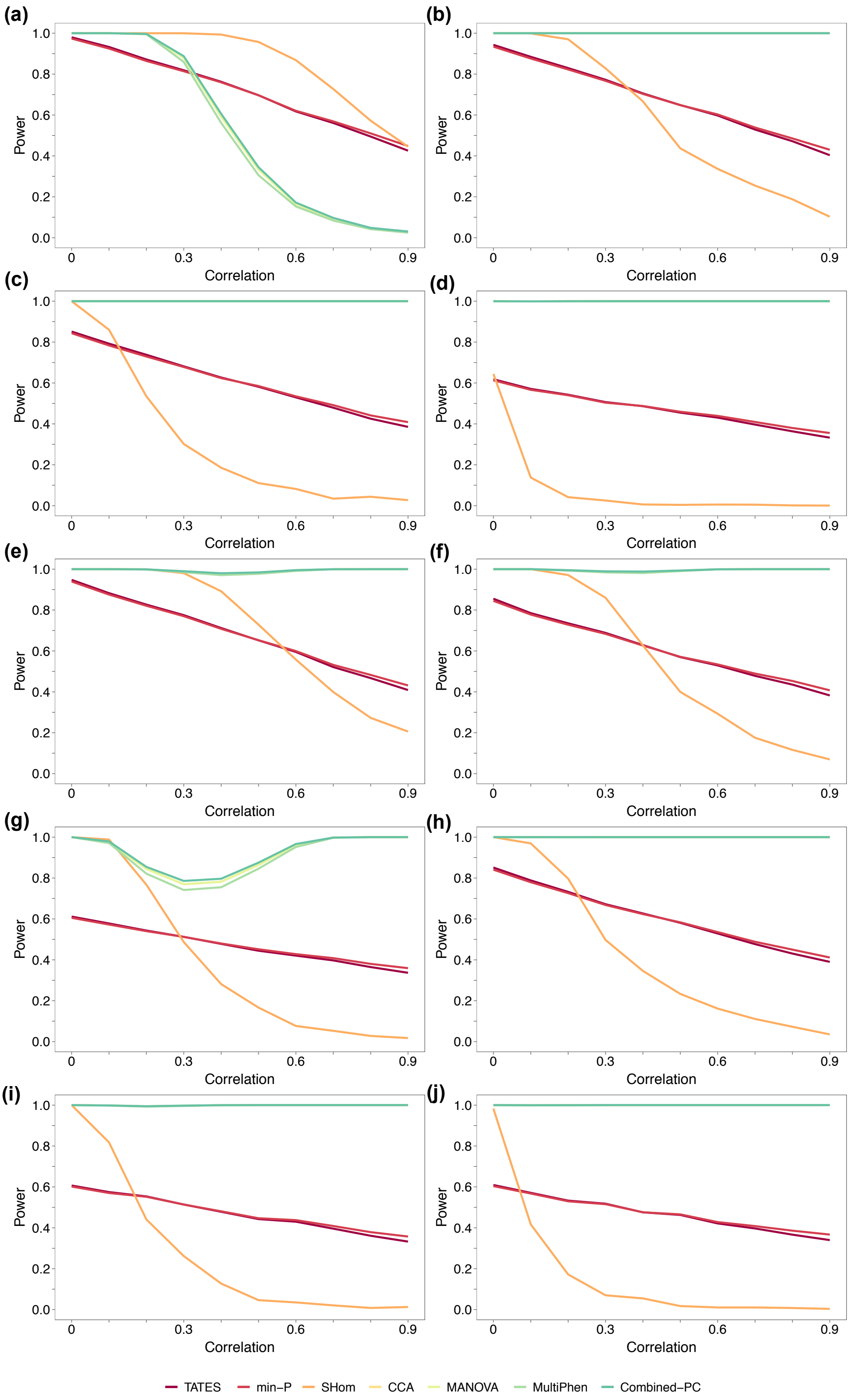

Supplementary Figure 3. Power comparisons from simulations of scenario 1 (S1), based on $v_1 - v_{10}$ (see **Table 2**) applied to data on 20 phenotypes, **(a)** – **(j)** respectively. The pairwise phenotypic correlations are the same for all phenotypes. Correlations < 0 are not possible across 20 phenotypes, hence the truncation in these results across the correlation range. mv-BIMBAM was not computationally feasible, and mv-SNPTEST not hard-coded, for 20 or more phenotypes and so were excluded here. S_{Het} is excluded, as a gamma distribution could not be estimated for these correlation matrices.

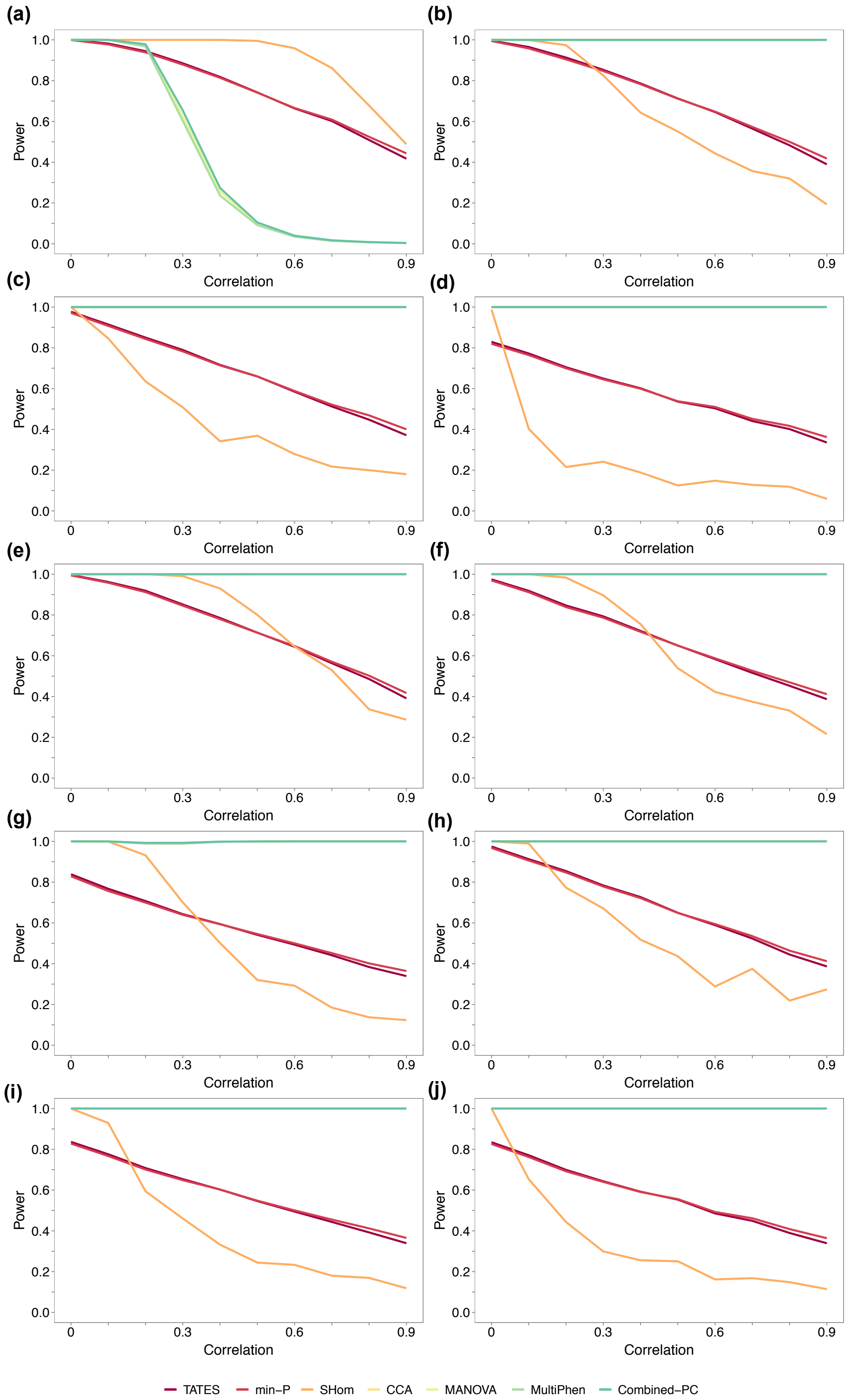

Supplementary Figure 4. Power comparisons from simulations of scenario 1 (S1), based on $\boldsymbol{v_1} - \boldsymbol{v_{10}}$ (see **Table 2**) applied to data on 48 phenotypes, **(a)** – **(j)** respectively. The pairwise phenotypic correlations are the same for all phenotypes. Correlations < 0 are not possible across 48 phenotypes, hence the truncation in these results across the correlation range. mv-BIMBAM was not computationally feasible, and mv-SNPTEST not hard-coded, for 20 or more phenotypes and so were excluded here. S_{Het} is excluded, as a gamma distribution could not be estimated for these correlation matrices.

S1: Null results for 2, 4, 8, 20 and 48 phenotypes

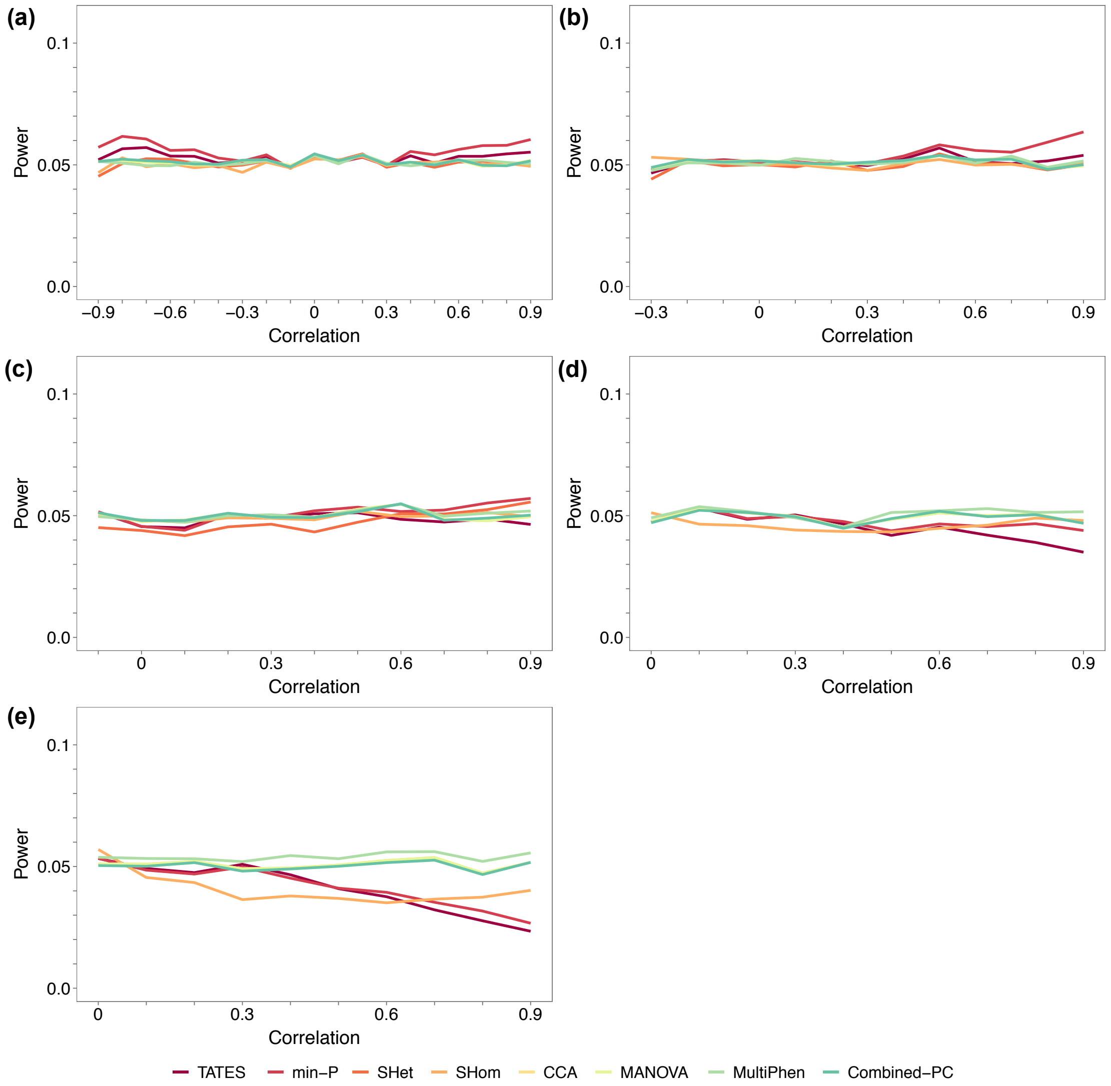

Supplementary Figure 5. Simulations of scenario 1 (S1) with direct effects under the null hypothesis of no genetic effect, applied to data on **(a)** 2, **(b)** 4, **(c)** 8, **(d)** 20 and **(e)** 48 phenotypes, based on $10{,}000$ replicates. The pairwise phenotypic correlations are the same for all phenotypes, and the genetic variants are simulated to explain zero variance in all phenotypes. S_{Het} is excluded for 20 and 48 phenotypes, as a gamma distribution could not be estimated for these correlation matrices.

S1: Results for 2 phenotypes with simulated downstream effect

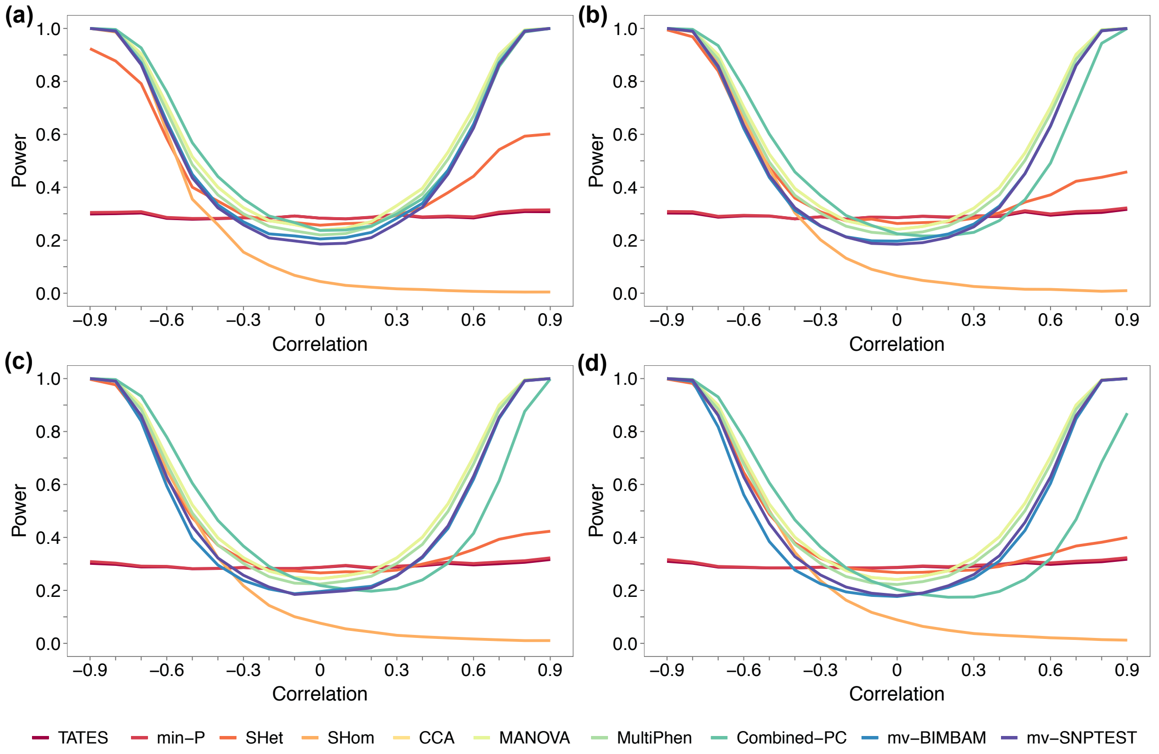

Supplementary Figure 6. Power comparisons from simulations of scenario 1 (S1) applied to data on two phenotypes with simulated downstream genetic effects. Phenotypic variance explained by the genetic variant in trait 1 is 0.5% in all cases, and in trait 2 is **(a)** 1%, **(b)** 5%, **(c)** 10% and **(d)** 20%. The pairwise phenotypic correlations are the same for all phenotypes.

S1: Null results for 2 phenotypes with simulated downstream effect

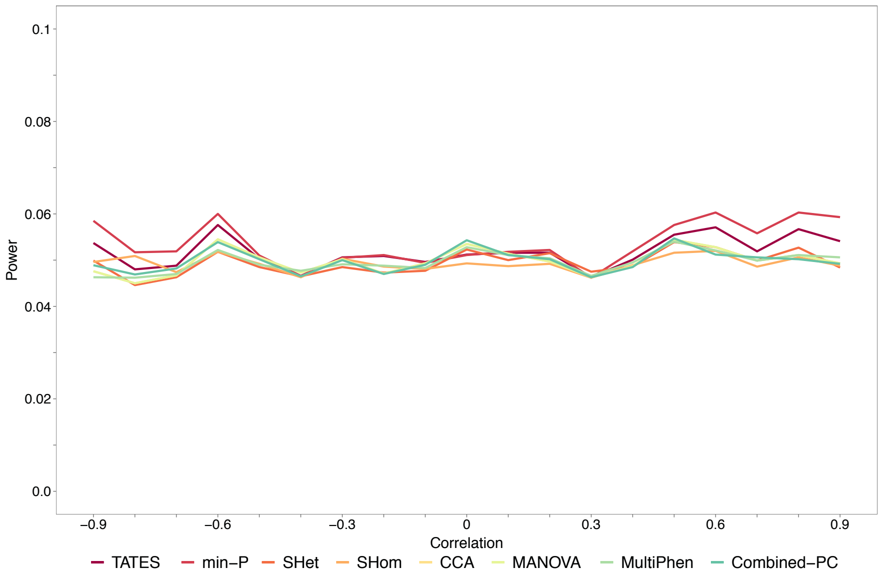

Supplementary Figure 7. Simulations of scenario 1 (S1) with downstream effects under the null hypothesis of no genetic effect, applied to data on 2 phenotypes based on 10,000 replicates. The pairwise phenotypic correlations are the same for all phenotypes, and the genetic variants are simulated to explain zero variance in the first phenotype, which has a downstream effect on the second phenotype.

S1: Power of methods applied to case/control data

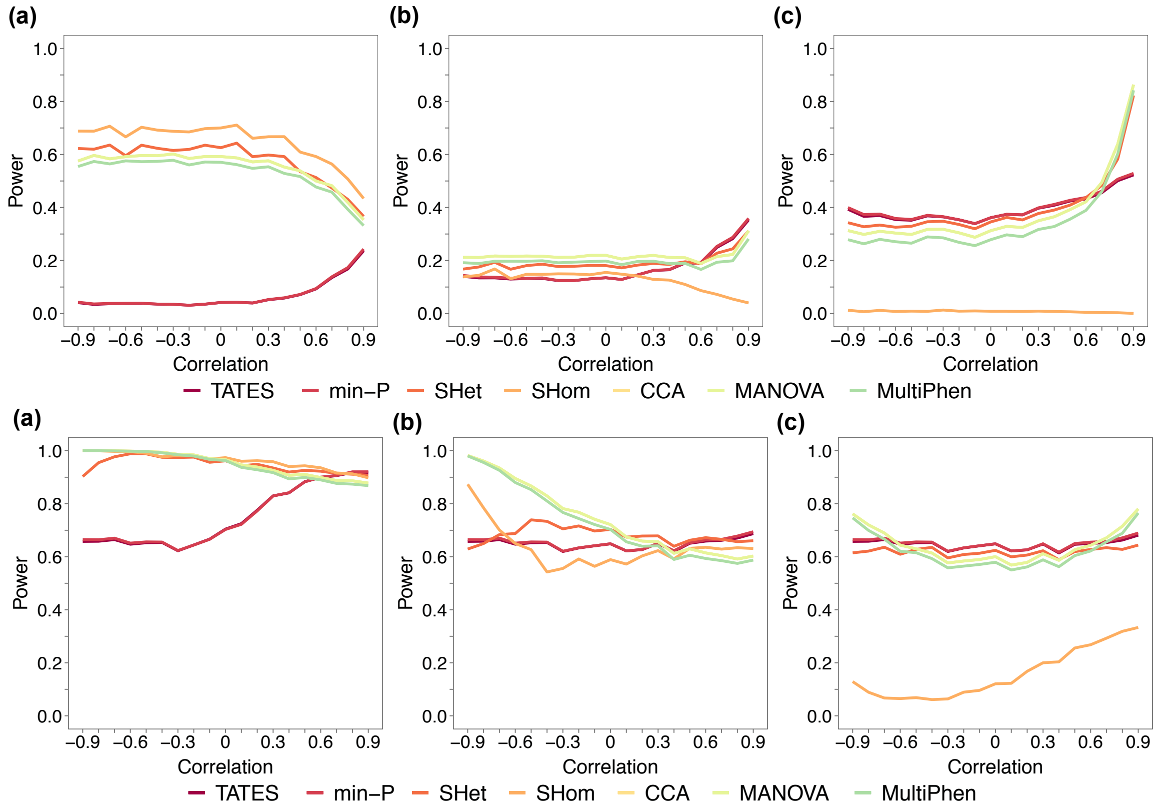

Supplementary Figure 8. Power comparisons for the simulations of scenario 1 (S1) involving two case/control phenotypes (top panel), and one case/control phenotype and a quantitative phenotype (bottom panel). The genetic variant either has **(a)** the same effect on both phenotypes, **(b)** a larger effect on the first phenotype, or **(c)** an effect on the first phenotype and no effect on the second – in the mixed phenotype scenarios the first phenotype is the quantitative phenotype (see **Methods** for details of these simulations). For all simulations, the case/control phenotypes have a simulated prevalence of 1% according to a liability threshold model.

S2: Results for 2, 4 and 8 phenotypes

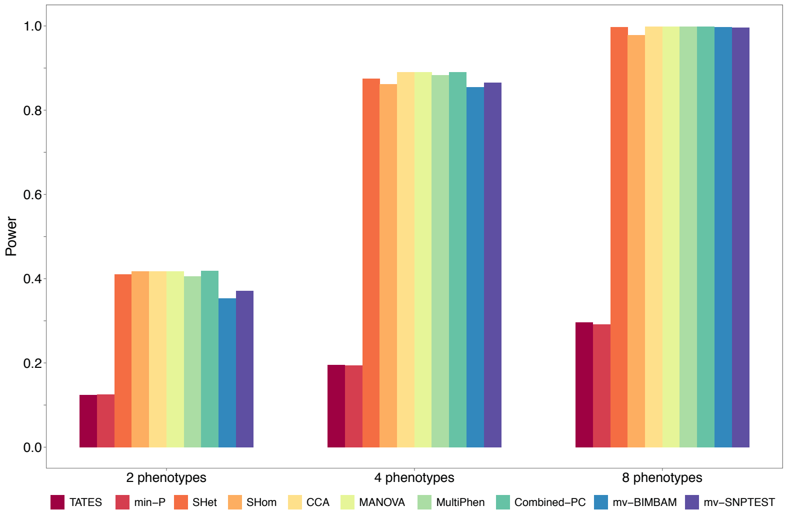

Supplementary Figure 9. Power comparisons for the simulations of scenario 2 (S2) involving 2, 4 and 8 phenotypes. In this scenario the phenotypic correlations are chosen uniformly such that the correlation matrix is positive definite, and the effect sizes are sampled uniformly between 0% and 0.5% phenotypic variance explained.

S3: Results for 2, 4, 8 and 48 phenotypes

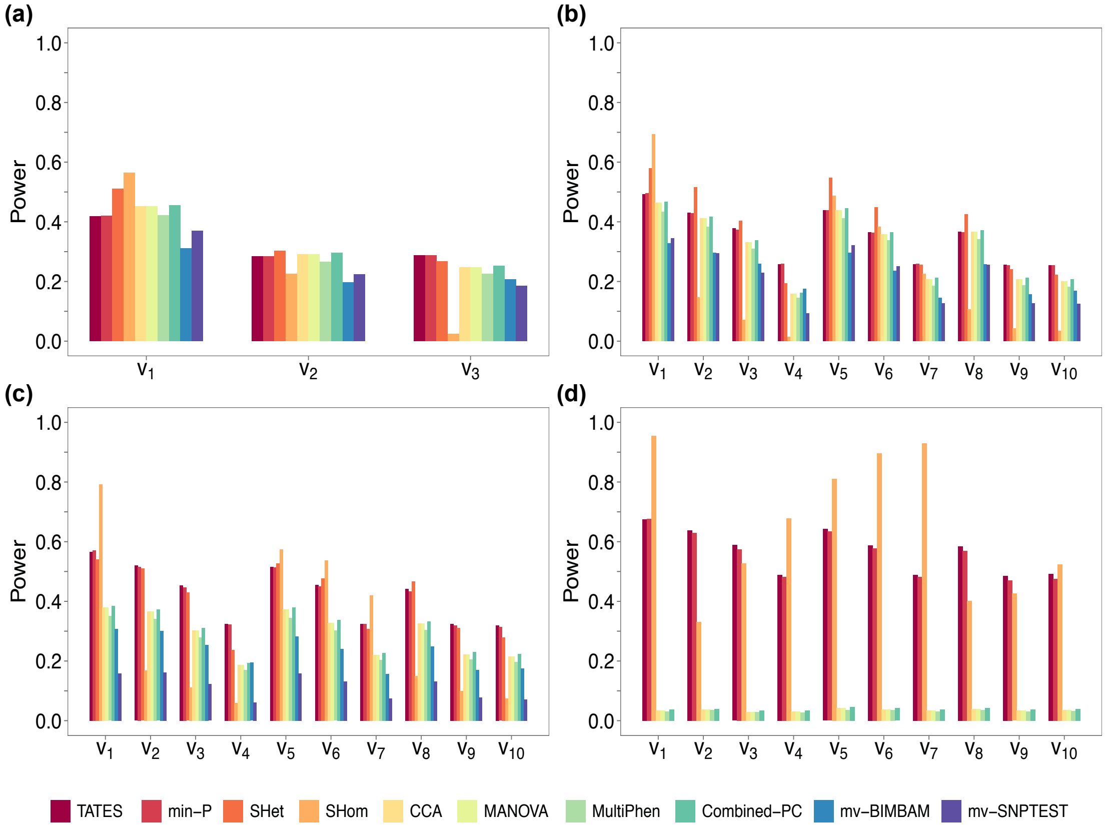

Supplementary Figure 10. Power comparisons for the simulations of scenario 3 (S3) involving **(a)** 2, **(b)** 4, **(c)** 8 and **(d)** 48 phenotypes. In this scenario the phenotypic correlations are chosen to reflect the relative genetic effect sizes defined in **Equations 2 – 4** for 2 phenotypes, and by the 10 genetic effect vectors (see **Table 2**) for 4, 8 and 48 phenotypes. mv-BIMBAM was not computationally feasible, and mv-SNPTEST not hard-coded, for 20 or more phenotypes and so were excluded here for 48 phenotypes. S_{Het} is excluded for 48 phenotypes, as a gamma distribution could not be estimated for these correlation matrices.

S4a: Phenotypic correlation density

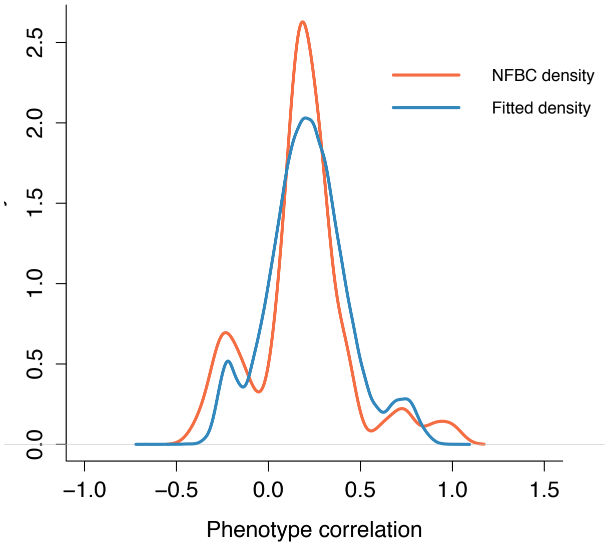

Supplementary Figure 11. Phenotypic correlation density based on 16 metabolic traits from the NFBC1966, and fitted mixture Gaussian density as given in **Equation 5** in **Methods**. Pairwise phenotypic correlations are sampled from this fitted density for scenario 4a (S4a).

S4a: Results for 2, 4 and 8 phenotypes

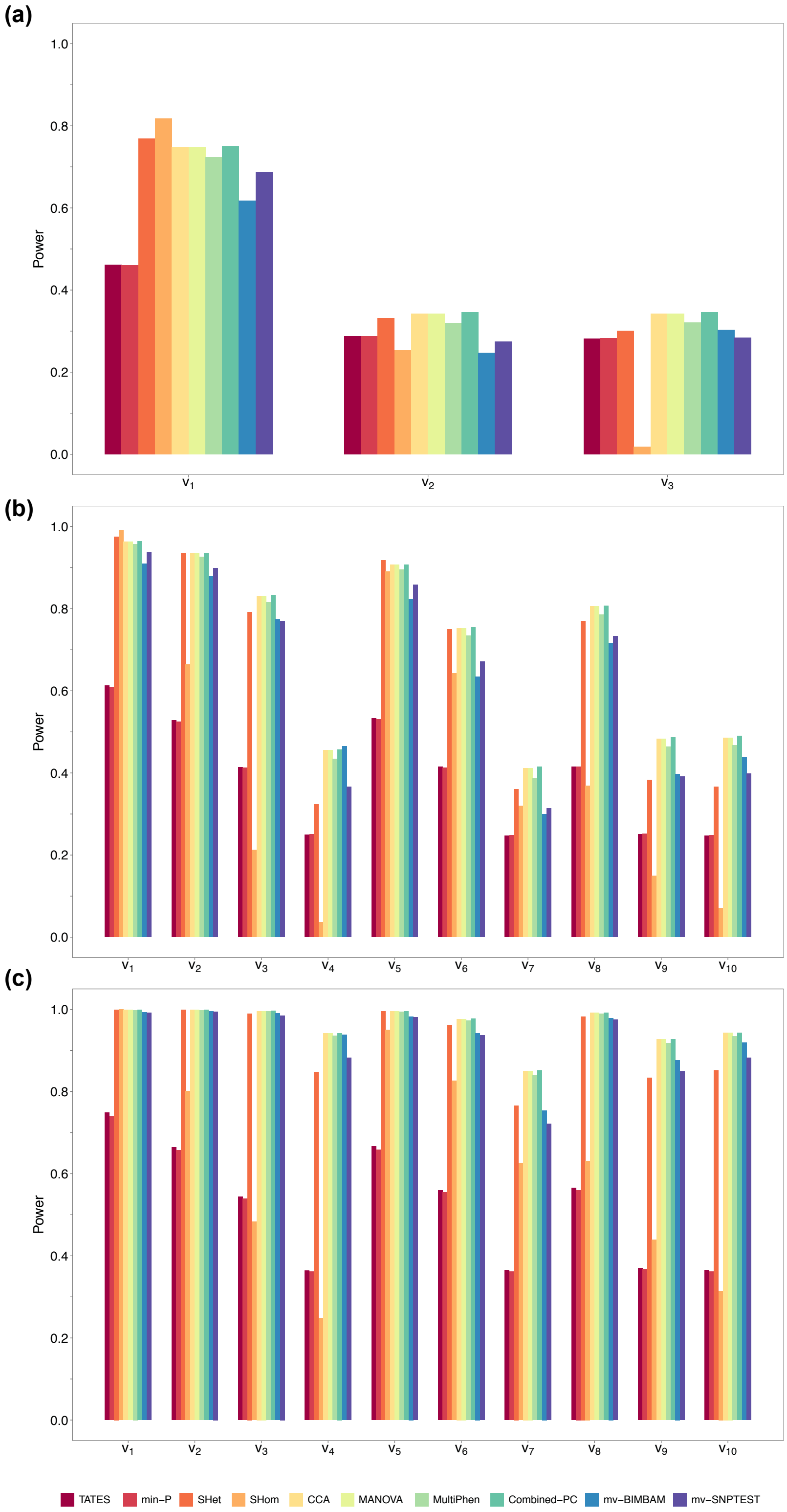

Supplementary Figure 12. Power comparisons for the simulations of scenario 4a (S4a) involving **(a)** 2, **(b)** 4 and **(c)** 8 phenotypes. In this scenario the phenotypic correlations are sampled from a fitted mixture Gaussian density (see **Equation 5** in **Methods**), and genetic effect sizes are defined by **Equations 2 – 4** for 2 phenotypes, and by the 10 genetic effect vectors (see **Table 2**) for 4 and 8 phenotypes.

S4b: Results for 2, 4 and 8 phenotypes

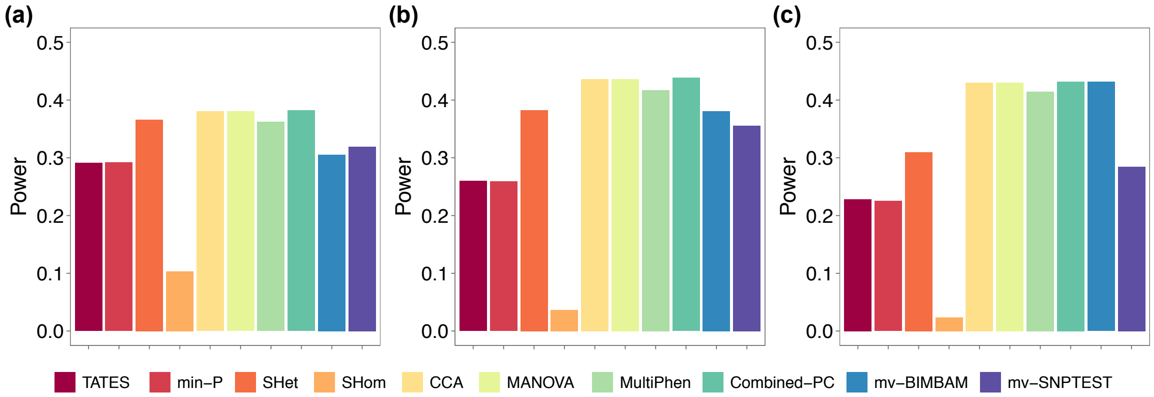

Supplementary Figure 13. Power comparisons for the real data informed simulations of scenario 4b (S4b) involving **(a)** 2, **(b)** 4 and **(c)** 8 phenotypes. In each case, data is simulated for all combinations of K phenotypes using the corresponding genetic effects and phenotypic correlations drawn directly from real data. The power results shown correspond to the average of the power estimates from all combinations.

S4b: Results for 2, 4, 8 and 12 phenotypes for S_{Het} on 10,000 samples

Number of phenotypes	Power for 5,000 samples	Power for 10,000 samples	Maximum individual-level method power	Relative increase over individual-level method
2	0.366	0.947	0.382	148%
4	0.382	0.940	0.439	114%
8	0.310	0.894	0.432	107%
12	0.210	0.835	0.432	93%

Supplementary Table 2. Power estimates for the S_{Het} method under simulation scenario 4b (S4b) with simulated data on $5{,}000$ and $10{,}000$ samples. The maximum power achieved by any individual-level data method for $5{,}000$ samples is shown, as well as the percentage increase in power for the S_{Het} method on $10{,}000$ samples compared to this individual-level data method on $5{,}000$.

Computation time estimates for the 10 multi-trait GWAS methods

Method	2 traits	4 traits	8 traits	12 traits
min-*P*	0.001	0.001	0.001	0.001
TATES	0.072	0.118	0.228	0.438
S_{Het}	4.612	8.127	15.437	26.497
S_{Hom}	0.006	0.008	0.007	0.008
CCA	0.967	1.211	1.534	2.156
MANOVA	1.257	1.468	2.127	2.979
MultiPhen	49.036	55.135	72.09	108.078
Combined-PC	3.421	5.922	11.567	20.912
mv-BIMBAM	6.72	16.763	1186.968	57050.64
mv-SNPTEST	25.779	39.421	68.478	120.066

Supplementary Table 3. Computation time estimates (in seconds) for the 10 methods for 2, 4, 8 and 12 phenotypes. We assessed the computation time for all 10 methods on a machine with a 2.7 GHz Intel Core i5 processor and 8 GB 1600 MHz DDR3 RAM. We simulated data for 5,000 samples, 100 SNP replicates with MAF 0.3, genetic variance explained of 0.5% for all phenotypes, and pairwise phenotypic correlations of 0.

S1: Results for highly sparse association signals

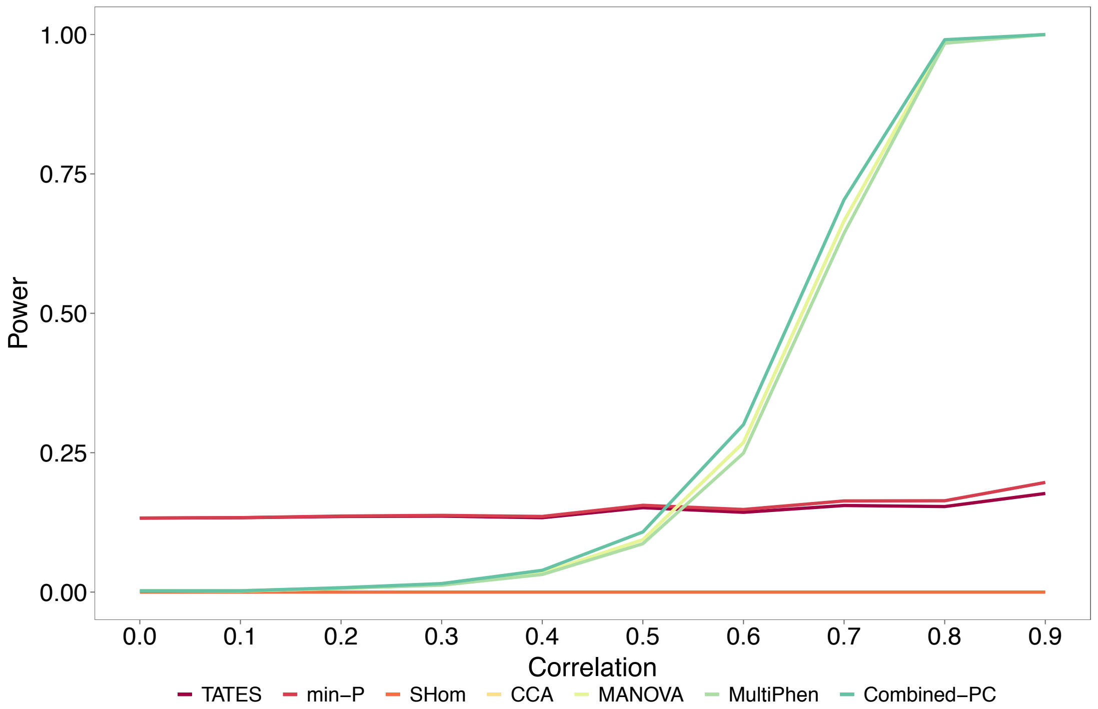

Supplementary Figure 14. Power comparisons from simulation of scenario 1 (S1) for 48 traits, where only one trait is affected by the genetic variant. The genetic variant is simulated to explain 0.5% variance in the affected trait. The pairwise phenotypic correlations are the same for all phenotypes. Correlations < 0 are not possible across 48 phenotypes, hence the truncation in these results across the correlation range. mv-BIMBAM was not computationally feasible, and mv-SNPTEST not hard-coded, for 20 or more phenotypes and so were excluded here. S_{Het} is excluded, as a gamma distribution could not be estimated for these correlation matrices.
